# Number of children and the prevalence of later-life major depression and insomnia in women and men: findings from a cross-sectional study of 0.5 million Chinese adults

**DOI:** 10.1186/s12888-020-02681-2

**Published:** 2020-05-29

**Authors:** Hanyu Wang, Minne Chen, Tong Xin, Kun Tang

**Affiliations:** 1 Research Center for Public Health, School of Medicine, Tsinghua University, Haidian District, Beijing, China; 2 School of Health Humanities, Peking University Health Science Center, No. 38 Xueyuan Road, Haidian District, Beijing, China; 3 Department of Sociology, University of North Carolina at Chapel Hill, 103 S Bldg Cb 9100, Chapel Hill, United States; 4 Department of Global Health, Peking University Health Science Center, No. 38 Xueyuan Road, Beijing, 100191 China

**Keywords:** Major depression, Insomnia, Parity, Sex differences, China

## Abstract

**Background:**

Pregnancy and parenthood have been associated with physical and mental health. Previous literature concerning the impacts of parity on mental health was inconsistent and lack epidemiolocal evidence. China, with growing mental health problems and changing fertility patterns, faces unique challenges. This study aims to examine the relationship between parity and the prevalence of major depression and insomnia among men and women in the Chinese population.

**Methods:**

Baseline data from a Chinese population-based study of 512,891 adults (59.01% women) from 10 areas, aged 30–79 were analyzed. Number of children was based on self-report by the participants. Major depression (MD) was assessed using the Composite International Diagnostic Inventory. Insomnia symptoms were accessed by a questionnaire comparable to that used in the Diagnostic and Statistical Manual of Mental Disorders. Logistic regression was used to assess the relationship between MD/Insomnia and number of children, after stratifications and adjustments.

**Results:**

For women, each additional child was associated with a 9% decreased odds of MD (OR: 0.91, 95%CI: 0.88–0.96), with the associations significant for those who lived in urban areas (OR: 0.76, 95%CI: 0.70–0.83), or had a lower education (OR: 0.90, 95%CI: 0.85–0.94), or had lower household income (OR: 0.89, 95%CI: 0.85–0.94), or had ever used alcohol (OR: 0.89, 95%CI: 0.84–0.93). The association between per additional children and MD was not significant in men (OR: 1.02, 95%CI: 0.97–1.07), but a decreased odd of MD with per additional child was found in men who lived in urban areas (OR: 0.81, 95%CI: 0.71–0.96). For women, each additional child was associated with a 4% decreased odds of insomnia (OR: 0.96, 95%CI: 0.95–0.96). Each additional child was also associated with a 2% decreased odds of insomnia in men (OR: 0.98, 95%CI: 0.97–1.00).

**Conclusions:**

MD and insomnia were inversely associated with number of children in women while the association was not overall significant in men. The association was mediated by socioeconomic and lifestyle factors. Future mental health public health programs should address parity and sex differences when designing interventions.

## Background

Pregnancy and parenthood have been associated with physical and psychological health through physiological and socioeconomic mechanisms in previous literature and parity were associated with both negative and positive effects on health [1–14]. Previous epidemiological research also showed that parity and parenthood were related to major diseases, including cardiovascular [1, 2], endocrine disease [3, 4], and cancer [5]. “J” shape association was observed between parity and cardiovascular disease in an American population [1] and diabetes in a Chinese population [3]. A positive association was found between number of children and coronary heart diseases and stroke in a Chinese population [2] and diabetes in a Danish cohort [4]. A negative association was found between parity and bladder cancer [5].

There was also a growing body of literature focusing on the psychological impacts of pregnancy, parity, and parenthood [6–14]. Physiological research found that the estrogen level was affected by women’s reproductive history and subsequently influenced mental health outcomes [15–17]. For example, estrone sulfate and parity were observed to be inversely associated parity [16]. Studies also found that early age at first birth was associated with adverse later-life mental health outcomes [6, 13]. Sociological studies also observed that number of children was related to mental health outcomes [10, 11, 13, 14]. A study conducted in 24 European countries observed that childlessness was associated with adverse mental health outcomes, with social pressure and gender mediating the association [10]. There was a positive relationship between number of children and later-life depression observed in Eastern Europe but not Western Europe and the association was affected by marital status and economic development of the countries [14]. A study in England also observed that more children were associated with worsened mental health among the older population and the association was mediated by wealth and lifestyle factors [13]. A recent study conducted in affluent areas in China found that more pregnancies were associated with a higher prevalence of depressive symptoms later in life [11]. Djundeva el al found that the association between children and later-life depression in Chinese elders was complicated by urbanization, gender preference, and modernization [12]. Psychological studies concluded that the association and pathways were highly complicated by life course, gender-related, socioeconomic, and cultural factors [7–9].

Previous literature, however, focused mostly on the psychological perspective of childrearing or physiological mechanisms of pregnancy rather than reproductive history and they are mostly without sound epidemiological evidence [6–10, 12, 15–17]; or explored the psychological, social perspective, including social capita or payback of having children with very limited medical interpretations [7, 13, 14]. Most studies were conducted in the western population [6–10, 13, 14] and the studies conducted in China had a small and limited sample, e.g. the sample is homogenous in terms of socio-demographic variants [11, 12]. Besides, there are very few epidemiological studies, if any, accessing the association between insomnia and parity.

The relationship between parity and mental status is particularly relevant to the Chinese population because of the rising mental health problems with poor mental healthcare, growing economic disparity, urban-rural migration, stressed working environment [18–20], the changing patterns of fertility behaviors [21], and the recently launched two-child policy [22]. As we discussed, previous studies in this area were mostly without sound epidemiological evidence, focusing on social support aspects, and conducting in the Western population. Besides, although insomnia is not generally considered as a psychiatry disorder itself, it is an important indicator of mental health and is a predictive risk factor for many mental illnesses [23]. Given that there was no specific study conducted in China to explore the association between parity and insomnia, this study can fill in this research gap. Thus, a population-based large-scale study exploring the association between number of children and later-life mental health outcomes in China is needed to close the gap in epidemiological literature and also provide evidence for policymakers and those who intended to improve mental health to make decisions and implement interventions.

This study aims to examine the relationship between parity and the prevalence of major depression and insomnia of half a million individuals from 10 diverse regions in China, with the consideration of socioeconomic and lifestyle factors. This study also examined the associations separately among women and men in this study population in order to differentiate biological and socioeconomic factors. In this study, we hypothesized that having more children might associate with better mental health outcomes, given that in the Chinese culture, more children were seen as blessed and more social support [24, 25]. The associations may differ between sexes and in different socioeconomic groups.

## Methods

### Sampling

The data were obtained from the baseline survey of a large cohort study conducted between 2004 to 2008 in 10 geographically defined areas (five urban and five rural areas). Details of the study design and sample characteristics could be found elsewhere [26]. The regional study sites were selected carefully to retain geographic and social diversity, and to maximize the difference in disease rates and risk exposure. More information concerning the study sites and selections can be found on the official website for this study [27]. Potential participants were approached by community health workers. Over 99% consented to participate in the baseline assessment. In total, 512,891 adults, including 302,632 (59%) women aged 30–79 years, approximately 30% of the total population of the 10 regions sampled, were recruited and completed interviewer-administered electronic questionnaire and clinic visits. A structured interview was conducted by community health workers during the clinical visit, covering socioeconomic factors, mental health related questions, and sleeping patterns. Physical metrics (e.g. height, weight, waist and hip circumference) were also recorded.

### Exposures

Number of children was based on the self-report by the participants. The question: “How many children do you have” was asked by the interviewers of the questionnaire and participants reported the current numbers of their children.

### Outcomes

In the present study, Chinese version of the computerized Composite International Diagnostic Inventory-Short Form (CIDI-SF) was employed to access major depression (MD). The evaluation was delivered face-to-face by trained community health workers at local clinics [28]. As a diagnostic instrument, the CIDI is based on criteria of the Diagnostic and Statistical Manual of Mental Disorders-IV (DSM-IV) which is proven to be generally equal to clinical psychiatric interviews [28]. The estimates of MD in the population level as accessed by the Chinese version of the CIDI were similar to those accessed by the Structured Clinical Interview for DSM [29, 30]. In the diagnostic tool, the participants with the presence of dysphoria and/or anhedonia accompanied by clustering of somatic, cognitive, and behavioral disturbances, including appetite or weight change, feelings of guilt or worthlessness, sleeping problems, fatigue, psychomotor changes, concentration problems, and thoughts of suicide that lasted 2 weeks or more were diagnosed as having MD [29, 30].

The present study employed a tool similar to that used in the Diagnostic and Statistical Manual of Mental Disorders (DSM-IV-TR) or Research Diagnostic Criteria (RDC) or International Classification of Sleep Disorders (ICSD) to access insomnia. During the interview, there were three questions to access insomnia: (1) taking > 30 min to fall asleep after going to bed or waking up in the middle of the night, (2) waking up early and not being able to go back to sleep and (3) needing to take medicine (including herbal or sleeping pills) at least once a week to help sleep. The interviewers asked the participants if they had any of the above three symptoms in at least 3 days or more in a week during the last month. If the participants answered ‘Yes’ to any of the above symptoms, then they were considered as having insomnia [31].

### Other co-variates

The covariates adjusted in the model were selected based on previous literature indicating that they are associated with the exposure and are independent risk factors for the outcomes. Demographic and socioeconomic characteristics collected in the baseline survey, specifically age at study date, sex, household size, the highest level of education, and occupation, were included as covariates in the analysis. Highest level of education was categorized into primary school/below and high school/above. Occupation was categorized into agriculture and related workers, factory workers, clerks, and unemployed and others. Participants’ health behaviors, including smoking habits and alcohol intake, were assessed by self-reported lifestyle status and classified as “never”, “ever” smoker/drinker. The reason that we classify smoke and alcohol use as “never” and “ever” instead of further dividing them is to allow a comparable sample size in each group. Participants’ health behaviors, including smoking status and alcohol use, were classified as “never,” “ever use”. BMI was calculated as weight in kilograms divided by height in meters squared [26]. BMI (kg/m^2^) was categorized as < 20, 20–25, and > 25 kg/m^2^, which was based on standard classification specific for the Chinese population [32].

Self-rated health was accessed by asking the participants: “How is your current general health status: excellent, good, fair, or poor?” in baseline interview and classified into four categories accordingly.

### Data analysis

Descriptive analyses were used to illustrate the basic demographic, socioeconomic and lifestyle factors for those people with no child, one child, two children, and more than two children. Means (SD) and percentages were presented as descriptive results. Logistic regression models were fitted to explore the association between number of children and MD and insomnia prevalence. Odds Ratios (ORs) and 95% CIs were calculated to explore the association between MD and insomnia by the number of children, stratified by sexes. One child group was chosen as the reference group for the analysis. Two types of logistic regression models were fitted: 1) crude 2) fully adjusted (adjusted for rural/urban, marital status, age at study date, level of attained education, household income, smoking status, alcohol use, self-rated health, occupation, BMI). To understand how rural/urban, age, education, household income, smoking, alcohol use, and BMI potentially modify the associations between number of children and MD/insomnia, adjusted sex-specific ORs and 95% CIs for MD and insomnia per additional child stratified by the variables described above were calculated, after adjusting for the study region, marital status, age at study date, highest education, household income, smoking status, alcohol use, self-rated health, occupation, BMI, where appropriate. All the data analysis was conducted by SAS version 9.4 (SAS Institute, Cary, NC, USA).

## Results

Detailed baseline characteristics of participants by number of children were presented in Table 1. Of all 512,891 participants included in the analysis, there were 10,300 with no child (2.01%), 183,628 with one child (35.80%), 166,394 with two children (32.44%) and 152,539 with more than two children (29.74%). The mean age of the study population for those with no child, one child, two children, and more than two children was 49.55 (SD = 12.04), 45.26 (SD = 7.37), 50.64 (SD = 9.34), and 60.18 (SD = 9.47) respectively. Compared to participants with one child, those with no or more than one child were generally older, less educated, had a lower household income, and generally had lower self-rated health. Those with one child were also less likely to have agriculture and related occupations (24.17%) compared with those with no child (39.96%) or more than one child (52.46% for those with 2 children and 51.26% for those with more than three children). Childless participants were much less likely to be married (51.08%) compared to those who had children. The prevalence of ever smoke was considerably higher in those with no child (51.86%) compared to those with children. Participants with no child (61.70%) or one child (62.73%) had a higher percentage of using alcohol, compared to those with two (51.42%) or more (46.26%) children. Detailed percentages and figures could be referred to in Table 1*.*

**Table 1:** Basic characteristics of participants

	Number of children			
	No Child	Children = 1	Children = 2	Children≥3
N, %	10,330, 2.01	183,628, 35.80	166,394, 32.44	152,539, 29.74
Socio-demographic characteristics				
Mean age, years (SD)	49.55 (12.04)	45.26 (7.37)	50.64 (9.34)	60.18 (9.47)
Female, %	40.21	57.60	59.73	61.18
Region is urban, %	48.06	64.91	31.88	32.11
Socio-economic characteristics				
Highest education, %				
Primary school / below	49.05	28.36	54.82	73.47
High school / above	50.95	71.64	45.18	26.53
Household income, %				
< 10,000yuan	69.23	44.5	56.53	72.71
≥ 10,000yuan	30.77	55.5	43.47	27.29
Occupation, %				
Agriculture and related	39.96	24.17	52.46	51.26
Factory workers	16.57	28.57	9.14	1.97
Clerk	18.23	26.08	10.71	4.49
Unemployed/Others	25.24	21.17	27.68	42.28
Marital status				
Married	51.08	94.30	94.18	84.87
Widowed/Divorced/Single	48.92	5.70	5.82	15.13
Lifestyle factors				
Self-rated health, %				
Poor	12.95	9.07	9.55	12.60
Fair	48.12	40.53	43.91	47.62
Good	22.53	28.59	30.13	25.76
Excellent	16.40	21.81	16.41	14.02
BMI (kg/m^2^), %				
< 20	20.99	10.29	12.58	16.13
20–25	54.61	56.17	55.52	53.27
> 25	24.40	33.55	31.90	30.60
Smoking, %				
Never	48.14	61.11	63.17	62.49
Ever smoke	51.86	38.89	36.83	37.51
Alcohol, %				
Never	38.30	37.27	48.58	53.74
Ever use	61.70	62.73	51.42	46.26

Table 2 presents the sex-specific association between number of children and MD/insomnia. The overall MD prevalence for females and males was 0.78 and 0.46%, respectively. The overall insomnia prevalence for females and males was 18.86 and 13.69%, respectively. In the fully adjusted model, the odds of MD is 1.27 times greater for those with no child (OR: 1.27, 95%CI: 1.00–1.62), compared to those with one child, and the association was only observed in women after stratified by sexes (OR:1.38, 95%CI: 1.03–1.87). Having two (OR: 0.95, 95%CI: 0.93–0.97) or more (OR: 0.91, 95%CI: 0.89–0.94) children were associated with lower odds of insomnia in the total population compared to those with one child and the association only existed in women (OR: 0.94, 95%CI: 0.91–0.96 for women with two children and OR: 0.88, 95%CI: 0.85–0.90 for women with three children) after stratification of sexes. The detailed ORs and 95% CIs could be referred to in Table 2.

**Table 2:** The association between number of children and major depression/ insomnia among women and men

	No Child		Children = 1		Children = 2		Children≥3	
	N %	OR	N, %	OR	N %	OR	N %	OR
***Total Population***								
*Depression*								
Crude Model	122 1.18	**1.97 (1.63,2.38)**	1108 0.60	1	1083 0.65	1.08 (0.99,1.17)	1027 0.67	**1.12 (1.03,1.22)**
Adjusted Model^a^		**1.27 (1.00,1.62)**		1		1.06 (0.97,1.16)		0.99 (0.88,1.10)
*Insomnia*								
Crude Model	1875 18.15	**1.38 (1.31,1.45)**	25,423 13.84	1	26,976 16.21	**1.20 (1.18,1.23)**	31,610 20.72	**1.63 (1.60,1.66)**
Adjusted Model^a^		1.00 (0.93,1.07)		1		**0.95 (0.93,0.97)**		**0.91 (0.89,0.94)**
***Female***								
*Depression*								
Crude Model	59 1.42	**1.90 (1.45,2.47)**	798 0.75	1	757 0.76	1.01 (0.91,1.12)	757 0.81	1.01 (0.91,1.12)
Adjusted Model^b^		**1.38 (1.03,1.87)**		1		0.97 (0.87,1.08)		0.88 (0.77,1.00)
*Insomnia*								
Crude Model	795 19.14	**1.31 (1.21,1.42)**	16,214 15.33	1	17,890 18.00	**1.21 (1.19,1.24)**	22,191 23.78	**1.72 (1.69,1.76)**
Adjusted Model^b^		0.93 (0.85,1.02)		1		**0.94 (0.91,0.96)**		**0.88 (0.85,0.90)**
***Male***								
*Depression*								
Crude Model	63 1.02	**2.58 (1.96,3.38)**	310 0.40	1	326 0.49	**1.22 (1.05,1.43)**	270 0.46	1.15 (0.97,1.35)
Adjusted Model^b^		1.09 (0.72,1.66)		1		**1.30 (1.09,1.54)**		**1.28 (1.04,1.58)**
*Insomnia*								
Crude Model	1080 17.49	**1.58 (1.48,1.69)**	9209 11.83	1	9086 13.56	**1.17 (1.13,1.21)**	9419 15.91	**1.41 (1.37,1.45)**
Adjusted Model^b^		1.05 (0.95,1.17)		1		0.98 (0.95,1.01)		0.96 (0.92,1.00)

^a^ Analyses were adjusted for sex, study region, marital status, age at study date, level of attained education, household income, smoking status, alcohol use, self-rated health, occupation, body mass index

^b^ Analyses were adjusted for study region, marital status, age at study date, level of attained education, household income, smoking status, alcohol use, self-rated health, occupation, body mass index

**Fig. 1:**
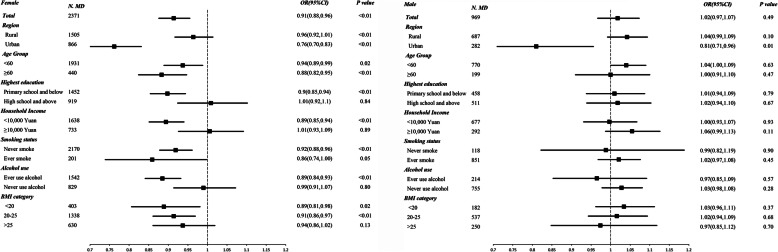
Adjusted odds ratios (95%CI)* for major depression per additional child by baseline characteristics in women and men^*^. * Analyses were adjusted for study region, marital status, age at study date, level of attained education, household income, smoking status, alcohol use, self-rated health, occupation, body mass index, where appropriate

Figure 1 presented the adjusted odds ratios for major depression per additional child by baseline characteristics in women and men. For women, each additional child was associated with a 9% decreased odds of MD (OR: 0.91, 95%CI: 0.88–0.96), with the associations significant for those who lived in urban areas (OR: 0.76, 95%CI: 0.70–0.83), or had a lower education (OR: 0.90, 95%CI: 0.85–0.94), or had lower household income (OR: 0.89, 95%CI: 0.85–0.94), or had ever used alcohol (OR: 0.89, 95%CI: 0.84–0.93). The association between per additional children and MD was not significant in men (OR: 1.02, 95%CI: 0.97–1.07) but a decreased odd of MD with per additional child was found in men living in urban areas (OR: 0.81, 95%CI: 0.71–0.96).

**Fig. 2:**
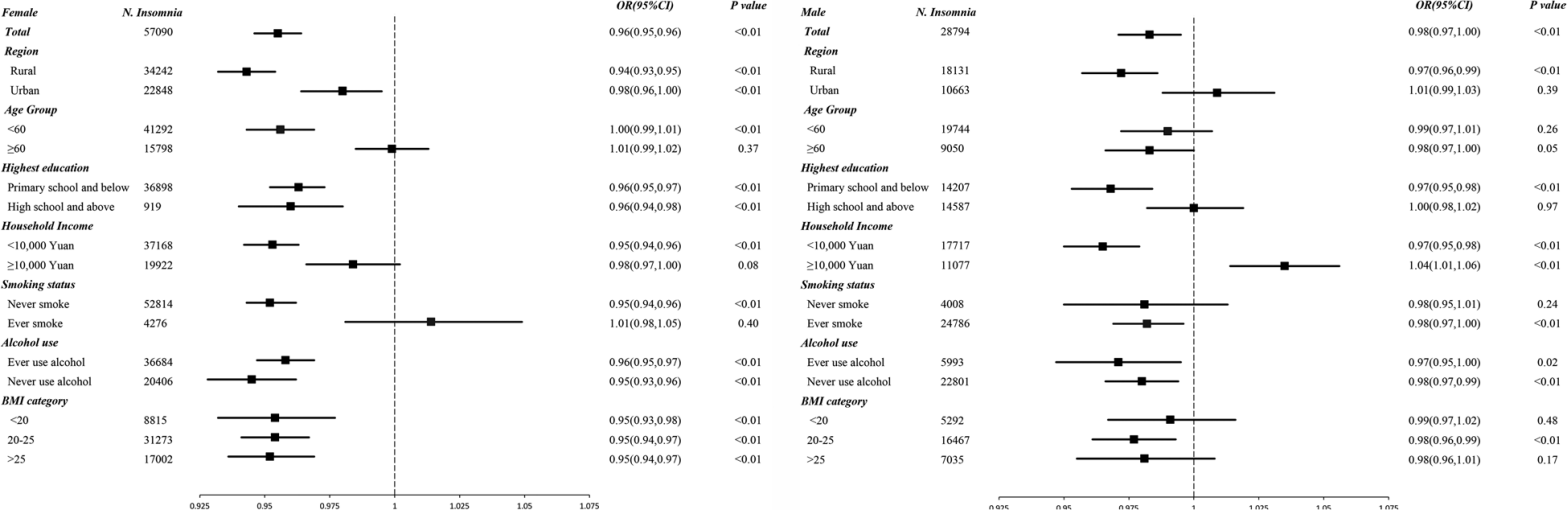
Adjusted odds ratios (95%CI)* for insomnia per additional child by baseline characteristics in women and men. * Analyses were adjusted for study region, marital status, age at study date, level of attained education, household income, smoking status, alcohol use, self-rated health, occupation, body mass index, where appropriate

Figure 2 presented the adjusted odds ratios for insomnia per additional child by baseline characteristics in women and men. For women, each additional child was associated with a 4% decreased odds of insomnia (OR: 0.96, 95%CI: 0.95–0.96), with the associations significant for those who lived in rural areas (OR: 0.94, 95%CI: 0..93–0.95), or aged less than 60 (OR: 0.96, 95%CI: 0.94–0.97), or had a lower household income (OR: 0.95, 95%CI: 0.94–0.96), or never smoked (OR: 0.95, 95%CI: 0.94–0.96). Each additional child was also associated with 2% decreased odds of insomnia in men (OR: 0.98, 95%CI: 0.97–1.00), with the household income appearing to significantly modify the association (OR: 0.97, 95%CI: 0.95–0.98 for those with household income< 10,000 Yuan and OR: 1.04, 95%CI: 1.01–1.06 for those with household income≥10,000 Yuan).

## Discussion

We found that number of children was associated with a decreased prevalence of depression in women but not in men. Number of children was also observed to be associated with decreased prevalence of insomnia in women and, less significantly, in men. Region appeared to have strong modifying effects of the associations while other socioeconomic status, including age, educational attainments, household income, and lifestyle factors (alcohol use and smoking behavior) also influenced the association.

In the present study, number of children was observed to be associated with better mental outcomes in women. Research exploring the association between number of children and later-life depression was scarce and the results were inconsistent with negative associations [33, 34], positive associations [11, 13, 14, 35], and no association [36] observed and the associations were complicated by hormones, socioeconomic, and lifestyle factors [11, 13, 14, 33–36]. In the present study, the trend of the association between mental outcomes and number of children was different among men and women. Thus, the physiological and social pathways are both possible. A recent study conducted in China found that having more birth was associated with increased risk of later-life depression for women and they hypothesized hormone level as the main mediator of the association [11]. Studies found that though endogenous estrogen level was high during pregnancy, women with more parity had lower circulating estrogen over a lifetime than those with fewer parity or nulliparity [15, 16] and estrogen generally had antidepressant effects [17]. In the Chinese culture in particular, however, more children and larger families were considered a blessing, which became especially true when one is older [24]. More children generally represented stronger social support, which is a strong contributor to better mental health outcomes [25]. It was possible that though the overall estrogen level increased the depression risk in later life, the positive cultural and social factors accompanied by more children overweighed the negative effects in the study population. The one-child policy may partially explain this association. Breaking the one-child policy sometimes meant a fine, which depended on the situation but often substantial [37]. Thus, the females choosing to have more children may be in a relatively well-off family, which lowers the risk of mental illnesses.

The associations between number of children and mental health outcomes were stronger in women than men. Firstly, the overall prevalence of major depression and insomnia was about 50% higher for women than men, which was consistent was previous literature [38]. The higher prevalence in the women sub-group made it more likely to demonstrate statistically significant results, which might partly contribute to the stronger association. While more birth was associated with low estrogen levels that increased depression risk [15–17], it was also possible that the social and cultural benefits of more children were much stronger for females than males. In traditional Chinese values, high fertility level was seen as a blessing for females [39]. At the same time, females in China are more likely to be in lower social strata than males [39, 40], which means that they need more support from children in later life. From the perspective of the one-child policy, studies have shown that this study empowered women in the society because of the indication of gender equality in this policy, but at the same time engaged more women into a highly competitive labor market and encouraged their immigration from rural to urban areas [41, 42]. The pressure of immigration and the modern work environment may increase the risk of women with only one child, which may serve as a potential explanation for the observed association.

The associations between parity and major depression and insomnia were observed to be influenced by region, among other socioeconomic and lifestyle factors. The protective effect of more children on major depression was significant in urban areas but disappeared in rural areas for both men and women. With the unprecedented economic growth, there has been a huge rural-urban migration of working-age adults in China [43]. This left middle-age and older people behind in rural areas, always alone, sometimes with grandchildren and living in a relatively deprived community [43, 44]. Previous studies have demonstrated that left-behind people in rural areas face a much higher risk of depression than their counterparts in urban areas [44, 45]. Moreover, previous research showed that middle-aged and older people in rural areas in China who lived in skipped generation households were observed to be less happy [44], which means that the pressure of raising grandchildren might also increase the risk of depression for people in rural areas. Thus, an additional child in rural areas might not bring actual benefits for the parents because the children migrated to urban areas and they also have to face the burden of raising the grandchildren.

This paper exhibits several strengths. First, although the data is cross-sectional, there is an inherent temporal effect between the parity and the time of the survey. Second, it is one of the first papers that examine the association between number of children and the prevalence of depression/insomnia in women and men simultaneously in China. In addition, the data we used is the largest cross-sectional study in China that incorporates data on depression and the number of children. We also included a range of socioeconomic variables, which allow us to explore the mediating effects and underlying pathways. The sex-segregated results generated by the present study might guide further mental public health policies.

The paper also has several limitations. First, the study is a cross-sectional study instead of a longitudinal study. While people’s situations may change with the elapse of time, longitudinal data would allow a better understanding of the causal pathways of number of children and subsequent mental health outcomes. Besides, the baseline study was conducted in 2007. The one-child policy has changed and socioeconomic structure in China improved in the recent decade. This study, however, can contribute to the ongoing research on the understanding of how changes in socioeconomic status and parity in the population affect mental health. Second, the underlying reasons for people to have children or not, or have how many children, were complicated by political, socioeconomic, as well as biological factors. There was no way for us to discover these underlying reasons, which might influence the associations observed in the present study. Lastly, depression and insomnia are complex physiological and psychological disorders, though we adjusted a wide range of socioeconomic and lifestyle factors, residual confounding by other factors, including political, social, or psychological determinants, could also influence the associations. It should also be noted that the overall prevalence of depression in the current study is relatively low (0.78% in females and 0.46% in males), which is below many estimates, especially those in western countries [46]. However, the prevalence of depression was complexed by many factors and varied by databases. The prevalence of depression presented in this research was verified by published research in the same database [47, 48].

## Conclusions

In the present study, number of children was negatively associated with the prevalence of major depression and insomnia. The associations were particularly significant for women. The associations were mediated by socioeconomic factors in general and region in particular. Public health interventions that aim at improving the mental health of middle-aged and older people should consider their number of children and related socioeconomic and lifestyle factors. The Chinese government announced in 2015 that China’s one-child policy has been lifted and is to be replaced by a universal two-child policy, which might alter the fertility pattern and subsequently influence people’s mental health. Further studies are needed to follow-up and identify the potential effects of the new policy on the well-being of the Chinese people.

## Data Availability

The data that support the findings of this study are available on request from the corresponding author.

## Abbreviations

MD: Major Depression
CIDI-SF: Composite International Diagnostic Inventory-Short Form
DSM-IV: Diagnostic and Statistical Manual of Mental Disorders-IV
BMI: Body Mass Index
ORs: Odds Ratios
SD: Standard deviation
